# Intratumoral CD56^bright^ natural killer cells are associated with improved survival in bladder cancer

**DOI:** 10.18632/oncotarget.26362

**Published:** 2018-11-23

**Authors:** Neelam Mukherjee, Niannian Ji, Vincent Hurez, Tyler J. Curiel, Maureen O. Montgomery, Andrew J. Braun, Marlo Nicolas, Marcela Aguilera, Dharam Kaushik, Qianqian Liu, Jianhua Ruan, Kerri A. Kendrick, Robert S. Svatek

**Affiliations:** ^1^ Department of Urology, University of Texas Health San Antonio (UTHSA), San Antonio, United States; ^2^ Department of Medicine, University of Texas Health San Antonio (UTHSA), San Antonio, United States; ^3^ Department of Pathology, University of Texas Health San Antonio (UTHSA), San Antonio, United States; ^4^ Department of Epidemiology & Biostatistics, University of Texas Health San Antonio (UTHSA), San Antonio, United States; ^5^ Department of Computer Science, University of Texas San Antonio (UTSA), San Antonio, United States

**Keywords:** bladder cancer, NK cells, tumor-infiltrating lymphocytes, CD56, survival

## Abstract

**Background:**

Natural killer (NK) cells are effective at killing tumors in a non-MHC restricted manner and are emerging targets for cancer therapy but their importance in bladder cancer (BC) is poorly defined. NK cells are commonly subdivided into populations based on relative surface expression of CD56. Two major subsets are CD56^bright^ and CD56^dim^ NK cells.

**Methods:**

The prevalence of intratumoral lymphocytes was examined via flow cytometric analysis of bladder tissue from a local cohort of patients with non-invasive and invasive BC (n=28). The association of NK cell subsets with cancer-specific survival (CSS) and overall survival (OS) was examined in 50 patients with BC using Cox regression. Fluorescence-activated cell sorting (FACS) of intratumoral lymphocytes isolated CD56 NK cell subsets were used for examination of function, including cytokine production and *in vitro* cytotoxicity.

**Results:**

NK cells predominated among bladder intratumoral lymphocytes. Intratumoral CD56^bright^ NK cells showed increased cytokine production and cytotoxicity compared to their CD56^dim^ counterparts and were associated with improved CSS and OS independent of pathologic tumor stage. On the other hand, CD56^dim^ NK cells were not associated with improved outcomes but were associated with higher pathologic stage.

**Conclusions:**

NK cells are frequent among intratumoral lymphocytes in BC. Bladder intratumoral CD56^bright^ NK cells are functional and prognostically relevant whereas CD56^dim^ NK cells are dysfunctional and prevalent in higher stage tumors. Thus, CD56^bright^ NK cells are promising targets in BC.

## INTRODUCTION

Examination of the phenotype and prognostic significance of intratumoral lymphocytes has led to important insights into disease immunopathogenesis and responses to immunotherapies. Bladder Cancer (BC) carries one of the highest mutational loads among tumors [1], which correlates with predicted tumor neo-antigen burden and response to immunotherapy [2]. Thus, BC is highly immunogenic and a relevant model to examine intratumoral immune compositions. Here, an unbiased examination of bladder tumor-infiltrating cells revealed a relative abundance of natural killer (NK) cells among lymphocytes, prompting further examination of their significance in BC.

The presence of intratumoral cytotoxic CD8^+^ T cells is associated with an improved prognosis in both non-muscle invasive [3] and muscle invasive BC [4], implicating these cells in BC control. To exert their cytotoxic function, CD8^+^ T cells require the presentation of antigens in the context of major histocompatibility complex class I molecules (MHC I) on the surface of tumor cells harboring mutated antigens. However, down-regulation of MHC I by tumor cells is an important mechanism by which tumors evade the immune system [5]. Under conditions of low tumor MHC I expression, other effector lymphocytes, especially NK cells, which mediate cytotoxicity via a non-MHC I restricted pathway, play an important role in anti-tumor defense [6]. NK cells have been classically described as innate immune effector cells involved in the first line of defense against infections and tumors [7] due to their ability to destroy target cells without antigen priming (as required by T cells) [8]. Although NK cells usually represent a minority of intratumoral lymphocytes, their presence correlates with improved survival in several other tumors, including lung, gastrointestinal, and head and neck [9]. NK cells are subdivided into subsets based on the relative surface expression of CD56 [10]. Two major subsets are CD56^bright^ and CD56^dim^ NK cells. Here, we define the functional properties and prognostic significance of these two subsets among bladder intratumoral NK cells.

## RESULTS

**Figure 1:**
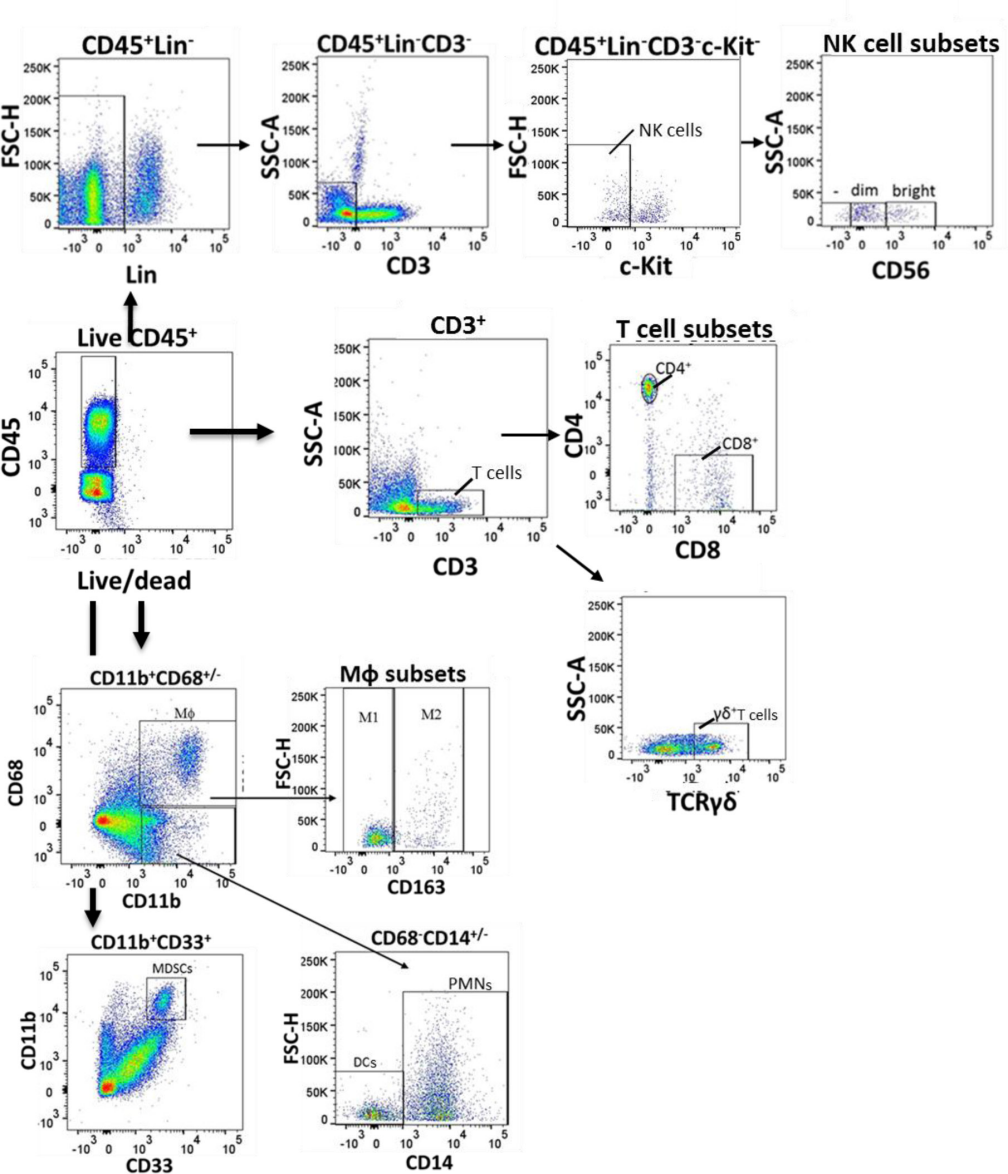
Flow gating of bladder intratumoral lymphocytes Human bladder tumor tissue was harvested and processed into single cell suspensions and analyzed with flow cytometry. Dot plots showing selected gating strategy as applied to the identification of major immune cell populations in tissue is a representative sample from among 28 patients. First, doublets were eliminated using a pulse geometry gate (FSC-A vs. FSC-H, not shown). Then, lymphocytes were gated on FVD (live/dead) and CD45. Natural killer (NK) cells were gated live CD45^+^ Lineage (CD14, ILT3, CD19)^-^CD3^-^ cKIT^-^ cells and were further characterized into CD56^bright^, CD56^dim^ and CD56^-^ populations. T cells were identified as live CD45^+^CD3^+^cells and the T cells were further characterized into CD4^+^, CD8^+^ and γδ T cells. Macrophages (MΦ) were identified as CD45^+^CD11b^+^CD68^+^ cells which were further divided into M1-(CD163^-^CD45^+^CD11b^+^CD68^+^ and M2 (CD163^+^CD45^+^CD11b^+^CD68^+^) macrophages. Myeloid derived suppressor cells (MDSCs) were identified as CD45^+^CD11b^+^CD33^+^ cells. Neutrophils (PMN) were identified as CD45^+^CD11b^+^CD68^-^CD14^+^ cells and dendritic cells (DCs) as CD45^+^CD11b^+^CD68^-^CD14^-^ cells.

**Figure 2:**
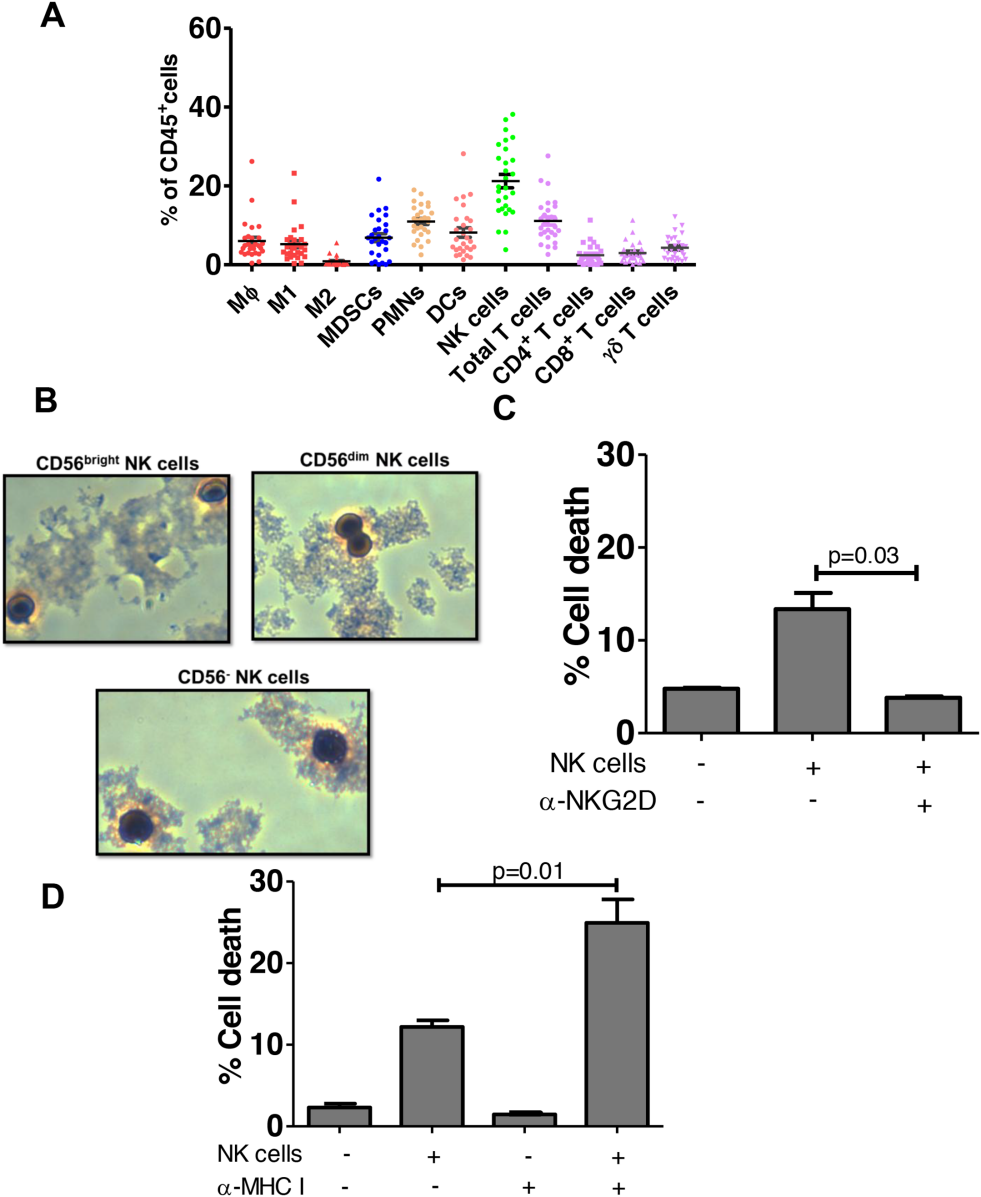
NK cells predominate among bladder intratumoral lymphocytes **(A)** Samples of human bladder tumors in local bladder cancer patient cohort (n=28) were taken at time of cystoscopy/cystectomy. Human lymphocytes were gated as in Figure 1 and plotted as % of CD45^+^ lymphocytes. MΦ- Macrophages, M1 – M1 macrophages, M2- M2 Macrophages, MDSCs- myeloid derived suppressor cells, PMN-polymorphonuclear cells (neutrophils), DC- dendritic cells, NK - natural killer, APC-Antigen presenting cells. Mean ± SEM. **(B)** Wright-Giemsa stain of CD56^bright^, CD56^dim^ and CD56^-^ NK cells isolated from bladder tumor tissue observed under light microscopy (40x magnification). **(C)** Cytotoxicity assay of sorted NK cells from human bladder tumor with CFSE-labeled K562 target cells in the presence of NKG2D-blocking antibody or an isotype control antibody. p-values represent two-tailed unpaired t-tests. **(D)** Graphed results of NK cell cytotoxicity assay with sorted NK cells from human bladder tumor with CFSE-labeled RT4 bladder cancer target cells. To block, MHC-I CFSE labeled target cells were incubated with MHC-I blocking antibody prior to addition of the NK cells. p-values represent two-tailed unpaired t-test.

We characterized intratumoral lymphocytes from patients with BC using comprehensive multi-parametric flow cytometry (population characteristics shown in Table 1, flow gating shown in Figure 1). NK cells (CD45^+^CD14^-^CD19^-^CD3^-^ILT3^-^cKIT^-^) were relatively frequent among intratumoral CD45^+^ lymphocytes (Figure 2A). To determine if these cells exhibit typical NK cell features, we electronically sorted them from bladder tumors and examined their morphology and cytotoxic capacity. Consistent with classic NK cell morphology, these bladder NK cells had condensed chromatin and cytoplasm containing large azurophylic granules (Figure 2B).

**Table 1:** Characteristics of patient cohort (n=50)

Median (IQR) follow-up time	15.0 (7.1-35.2) months
Mean (range) age	69 (45-86) years
Gender	
Female	11 (5.6%)
Male	39 (72.2%)
Race	
White	41 (82.0%)
African American	2 (4.0%)
Other/Unknown	7 (14.0%)
Ethnicity	
Hispanic	7 (14.0%)
Non-Hispanic	43 (86.0%)
Stage	
Tis/Ta	8 (16.0%)
T1	12 (24.0%)
T2	22 (44.0%)
T3	6 (12.0%)
T4	2 (4.0%)
Histologic Subtype	
Pure urothelial	45 (90.0%)
carcinoma	5 (10.0%)

NK cell cytotoxicity is modulated by activating and inhibiting receptors expressed on their cell surface [11]. Binding of inhibitory receptors by ligands such as MHC I, results in inhibition of NK cell mediated lysis [11]. On the other hand, ligand binding of the activating receptor Natural Killer Group 2D (NKG2D) stimulates NK cells and induces cytotoxicity [12]. NKG2D recognizes a large number of activating ligands, which are structurally related to the MHC molecule, including MHC class-I-chain related protein A (MICA), MHC class-I-chain related protein B (MICB), and UL16-binding proteins (ULBP1-6) [13]. We found bladder derived NK cells were cytotoxic against NK cell-sensitive K562 cells (Figure 2C) and blocking the activating receptor NKG2D, diminished bladder NK cell cytotoxicity (Figure 2C), validating them as bona fide NK cells [14]. To determine if bladder tumor MHC I expression affects NK cell cytotoxicity *in vitro*, we examined NK cell cytotoxicity against RT4 BC cells which express relatively more MHC I than K562 cells but also express NKG2D activating ligands MICA, MICB and ULBP1, 2 and 3 (Supplementary Figure 1). Consistent with the notion that bladder tumor MHC I inhibits NK cell cytotoxicity [15], *in vitro* blocking of MHC I significantly increased NK cell-mediated cytotoxicity against RT4 cells (Figure 2D). Together, these data suggest that under conditions of low tumor MHC I, the inhibitory signals mediated by MHC I to NK cells are decreased and NK cells are more cytotoxic.

NK cells are commonly divided into two developmentally related, but functionally distinct populations based on surface expression of CD56: CD56^bright^ and CD56^dim^ NK cells^10^. Published reports of NK cell subsets describe mutual exclusivity in regards to function with one subset having relatively higher cytokine production and lower cytotoxicity compared to the other subset^10^. Generally, CD56^bright^ NK cells exhibit enhanced IFN-γ cytokine production and decreased cytotoxicity compared to their CD56^dim^ counterparts [16]. However, most work to date has examined NK cells isolated from peripheral blood and less is known about intratumoral NK cells.

**Figure 3:**
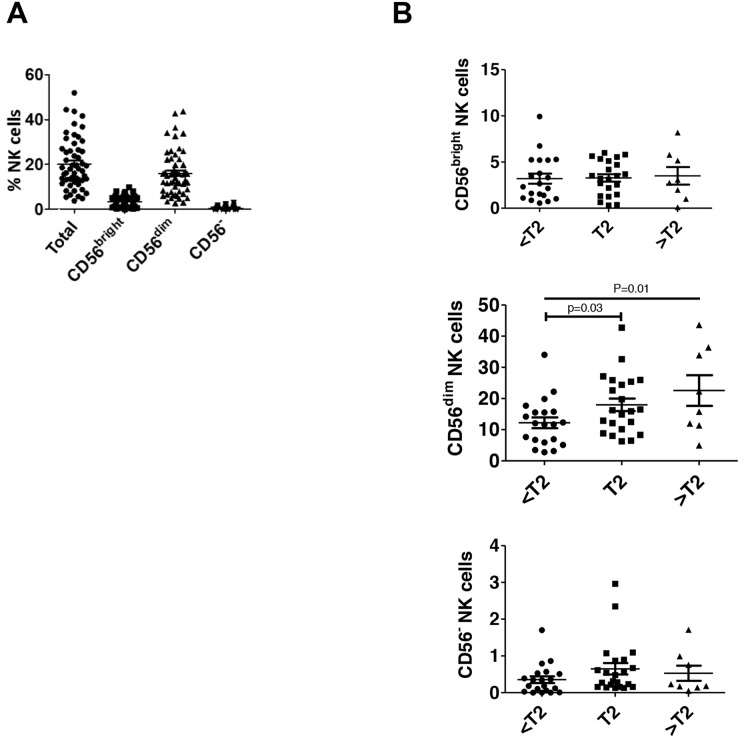
CD56^dim^ NK cells are increased in higher stage bladder tumors Human bladder tumor samples from n=50 patients were processed into single cell suspensions and analyzed with flow cytometry as in Figure 1. **(A)** Plotted total and NK cell subsets as a percentage of intratumoral live CD45^+^ lymphocytes. Mean ± SEM. **(B)** Plotted NK cell subsets as a percentage of intratumoral live CD45^+^ lymphocytes across pathologic tumor stage. Mean ± SEM, p-values represent two-tailed unpaired t-test and posttest for linear trend.

**Figure 4:**
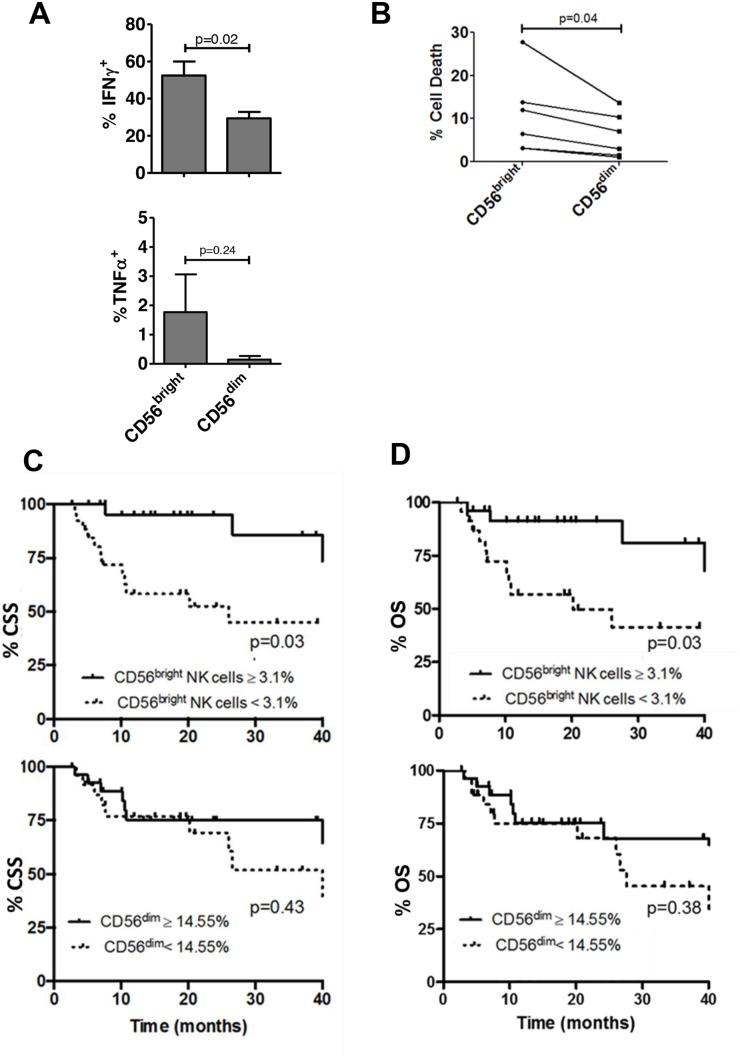
Intratumoral CD56^bright^ NK cells are more functional than CD56^dim^ NK cells and correlate with survival in bladder cancer Bladder intratumoral NK cells were characterized as CD56^bright^ and CD56^dim^ populations using flow cytometry. **(A)** Proportion of NK cells with cytokine or perforin production was identified by flow cytometry. IFN-γ^+^ and TNF-α^+^ cells are shown as a percentage of CD56^bright^ or CD56^dim^ NK cells. p-values represent two-tailed unpaired t-test. **(B)** *in vitro* cytotoxicity assay of intratumoral NK cells sorted from human bladder tumor tissue against K562. % cell death for CD56^bright^ and CD56^dim^ subsets were calculated for 6 representative patients. p-value represents paired two-tailed t test. Kaplan-Meier plots of **(C)** cancer specific survival (CSS) and **(D)** overall survival (OS) of bladder cancer patients according to intratumoral CD56^bright^ and CD56^dim^ NK cells. p-values represent log-rank (Mantel-Cox) test.

To examine bladder NK cell subsets, intratumoral NK cells were sorted into one of three different groups based on CD56 surface expression including CD56^bright^, CD56^dim^, and CD56^-^ NK cells (Figure 1). In bladder tumors (Figure 3A), the majority of NK cells were CD56^dim^ representing approximately 75% of total bladder NK cells followed by CD56^bright^ (~14%) and CD56^-^ (~2%). No difference in morphology was observed between the CD56 subsets in bladder tumors (Figure 2B). Intratumoral CD56^-^ NK cells were rare and not associated with pathologic stage, CSS, or OS (not shown). Intratumoral CD56^dim^ NK cells had higher surface expression of the Fcγ receptor IIIA CD16 (Supplementary Figure 2) compared to CD56^bright^ NK cells, consistent with the well-characterized association of CD16 expression in the CD56^dim^ subset among circulating NK cells [17]. The proportion of intratumoral CD56^dim^ NK cells increased in higher stage tumors, whereas the proportion of CD56^bright^ NK cells remained unchanged across pathologic stages (Figure 3B). Remarkably, unstimulated intratumoral CD56^bright^ NK cells produced more IFN-γ (Figure 4A) and were more cytotoxic than intratumoral CD56^dim^ NK cells (Figure 4B). This supports novel functional characteristics of NK cells in the bladder tumors that lack mutual exclusivity of function previously described for CD56 NK cell subsets [10].

Given functional differences observed between bladder NK cell subsets, we questioned whether the prognostic significance of NK cells varied across these CD56-defined NK cells. As predicted by their improved function relative to CD56^dim^ subset, intratumoral CD56^bright^ NK cells were associated with improved CSS and OS whereas no association with survival outcomes was seen with the CD56^dim^ population (Figure 4C-4D). Importantly, the association of intratumoral CD56^bright^ NK cells with survival remained significant on multivariable analysis that included pathologic stage (Table 2). Together these findings support distinct phenotypes of bladder intratumoral NK cells across CD56 expression with intratumoral CD56^dim^ associated with increased pathologic stage and intratumoral CD56^bright^ NK cells associated with more favorable outcomes.

**Table 2:** Bladder intratumoral CD56^brigh^^t^ NK cells are independently associated with decreased overall mortality and cancer-specific mortality

	Overall mortality			Cancer-specific mortality		
Variable	HR	95% CI	P	HR	95% CI	P
CD56^bright^ NK cells	0.36	0.14 - 0.92	0.03	0.27	0.09 - 0.78	0.02
Pathologic stage	3.12	1.47 - 6.63	<0.01	3.73	1.64 - 8.51	<0.01

## DISCUSSION

In this study, examination of bladder intratumoral lymphocytes showed that NK cells were prevalent among intratumoral lymphocytes in BC and the CD56^bright^ NK cells were associated with improved survival outcomes independent of pathologic tumor stage. These findings support prognostic relevance of intratumoral NK cells in BC and strategies to boost NK cell numbers and/or function in BC as rational approaches.

Our findings of functional characteristics of intratumoral NK cells challenge the dogma of mutual exclusivity of function previously described for NK cell subsets. Surface expression of CD56 is commonly used to define distinct populations of NK cells [18] but NK cells subset functionality is contextual dependent and influenced by many factors including both soluble molecules and receptor binding ligands [19]. The current doctrine holds that CD56-defined NK cells subsets have either high cytotoxicity coupled with low cytokine production or low cytotoxicity coupled with high cytokine production [8, 10]. Generally, higher cytotoxic effector proteins and increased cytotoxicity is attributed to the CD56^dim^ subset whereas CD56^bright^ produce higher level of effector cytokines but generate ineffective anti-tumor responses [10, 17]. In bladder tumors, however, CD56^dim^ NK cells are both poorly cytotoxic and produce fewer cytokines compared to CD56^bright^ NK cells.

The CD56^bright^ NK cell population is generally considered an immature precursor of the CD56^dim^ subset based on the linear differentiation model of NK cells [20]. However, other reports suggest that activated CD56^dim/bright^ cells can modulate their CD56 expression [21]. Thus, these two different NK cell phenotypes could represent sequential differentiation populations or terminally differentiated populations that shuffle between each other as a result of external pressures from surrounding tumor microenvironment. BC, we speculate that higher stage tumors, modulate NK cells, inducing a more dysfunctional NK cell, marked by loss of CD56 surface expression. We speculate that immunosuppressive conditions in more advanced tumors partially contributes to the increase in the CD56^dim^ population as these NK cells were found to be less functional and poorly prognostic compared to their CD56^bright^ counterparts. This concept is supported by work showing that NK cells exposed to immunosuppressive environments down-regulate their CD56 expression and become less functional [22].

Control of NK cells effector activity by activating and inhibitory receptors is well established but the role of these receptors in human intratumoral NK cells is not well studied. MHC I is normally present on the urothelial cell surface, but bladder tumors can downregulate its expression [23]. Sharma and colleagues [4] observed an association between clinical outcome and tumor MHC I expression in patients undergoing cystectomy; patients with low compared to high MHC I had a median CSS of 20 versus 57 months, respectively (p=0.09). Conventional T cell immune therapy, including antibodies against PD-L1 and PD-1, are currently used for advanced bladder cancer, and are thought to depend on tumor-antigen presentation by an intact MHC I receptor. Because NK cells could elicit BC cytotoxicity in the absence of MHC I, targeting NK cells is an attractive strategy to complement T cell based methods in BC.

We previously described how aging does not simply decrease immune cell populations but rather, promotes potentially detrimental changes in immune functions [24]. Decreased frequency or function of NK cells can reduce the efficacy of many biologic processes including immune surveillance and tumor elimination [25]. As BC is strongly associated with aging and NK cells are prognostic in BC, we speculate that age-related decreases in NK cell frequency or function could contribute to BC development. Although not reaching statistical significance, CD56 expression on NK cells correlated inversely with age (Supplementary Figure 3A). In addition, the proportion of intratumoral CD56^bright^ but not CD56^dim^ decreased across age of patient (Supplementary Figure 3B). These findings suggest that aging could promote dysfunction in NK cells, scored by fewer bladder intratumoral CD56^bright^ NK cells, increased CD56^dim^ NK cells and potentially contributing to increased BC prevalence in older populations. Supporting this notion, aging is associated with increase in CD56^dim^ and decrease in CD56^bright^ NK cells in peripheral blood [26].

This study has important limitations. The cohort size is modest. As a result of limited events, the number of parameters included in multivariable analyses was constrained. Nevertheless, our cohort is relatively large among published studies describing comprehensive characterization of intratumoral lymphocytes with flow cytometric methodology [27, 28]. By including patients with both non-muscle invasive and muscle-invasive BC, the study is less homogenous but captures a broad spectrum of disease risk. The study is subject to inherent challenges to acquisition of tissue from patients undergoing surgery, including variability in surgical technique and time between tissue removal and processing, which could systematically influence the lymphocyte content and function. Also, the gating strategy is subject to criticism because a uniformly agreed upon standard strategy for identifying NK cells does not exist. Despite these limitations, this study provides novel findings using a comprehensive analysis of intratumoral NK cells in BC with a highly selective gating strategy for NK cells compared to published methods [10, 17].

## MATERIALS AND METHODS

### Immune cell phenotyping and data from local BC cohort

Patients were recruited through a local Institutional Review Board (IRB) approved observational cohort study, which collected clinical data and bladder tissue for analysis (IRB # BCR20120159H). Eligible patients were 18 years of age or older and had a confirmed or suspected diagnosis of BC. All patients provided written informed consent. Patient demographics, pathology and imaging reports, physical exam and laboratory assessments, and specimen tracking data were entered prospectively into a secured web-based REDCap database system. This study's involvement with human subjects complies with the Declaration of Helsinki.

Bladder tumors were surgically excised under sterile conditions as per standard-of-care. A portion of the tumor was separated and placed in Roswell Park Memorial Institute (RPMI) 1640 medium containing 1% antibiotic (Penicillin-Streptomycin) and transported on ice. Fresh tumor tissues were washed with phosphate buffered saline (PBS) and minced into 1-2 mm pieces and incubated in digestion solution (1mg/ml collagenase, 0.25% trypsin and 0.25mg/ml DNAse) for 40 minutes at 37°C, 5% CO_2_. After digestion the enzymes are neutralized by addition of complete RPMI containing 10% fetal bovine serum (FBS) and the samples were filtered through 100 μM filter to produce single cell suspensions. Single cell suspensions were stored in -150°C until use.

Intratumoral cells were stained and analyzed or sorted as previously described [29], using LSR II and Fluorescence-activated cell sorting (FACS) Aria II flow cytometers and FACSDiva software (BD Biosciences). Compensation was performed with single color controls prepared using BD Biosciences Comp Beads. Single cells suspension was mixed with Brefeldin A as Golgi blocker for 5 hrs. before cytokine staining. Compensation matrices were calculated automatically and sample analysis was carried out using FACSDiva software and FlowJo software. Antibodies and dyes used in flow cytometry are as follows: Fixable Viability Dye eFluor™ 455UV (FVD) (eBioscience™); Anti-human antibodies: CD45 (Clone: HI30); CD14 (Clone: M5E2); CD85k (ILT3) (Clone:ZM4.1); CD19 (Clone: HIB19); CD3 (Clone: SK7); CD117 (c-kit) (Clone:104D2); CD56 (NCAM) (Clone:5.1H11); CD16 (Clone: 3G8); CD3 (Clone: UCHT1); CD4 (eBioscience™, Clone:OKT4); CD8 (Clone: SK1); γ/δ (Clone: B1); CD11b (eBioscience™, ICRF44); CD68 (BD Horizon™, Clone Y1/82A (RUO); CD33 (Clone: B1); CD163 (Clone: GHI/61); CD14 (Clone: HCD14); IFN-γ (Clone: 4S.B3); and TNF-α (Clone: MAb11). Representative gating strategy for immune cells is shown in Figure 1. NK cells were identified and sorted by their cell surface markers (CD45^+^CD14^-^CD19^-^CD3^-^ILT3^-^cKIT^-^) [30–32] and further divided into CD56^bright^, CD56^dim^ and CD56^-^ populations. Light microscopy (40 x magnification) was used to visualize flow-sorted NK cells stained with Wright/ Giemsa and the staining was performed as described in [33].

### Cell culture

RT4 and T24 human BC cells were cultured in McCoy's 5a Medium supplemented with 10% FBS.UM-UC3, UM-UC6 and UM-UC14 human BC cell lines were cultured in Minimum Essential Medium (MEM) with FBS (10%), sodium pyruvate (1%), non-essential amino acids (0.1%), sodium bicarbonate (2%). SCC4 human squamous cell carcinoma cells were cultured in Dulbecco's Modified Eagle Medium: Nutrient Mixture F-12 (DMEM:F12) Medium supplemented with 400 ng/ml hydrocortisone and 10% FBS. K562 (chronic myeloid leukemia cells) were cultured in RPMI supplemented with 10% FBS and 2 mM *L*-glutamine. All cells were grown under standard conditions (37°C, 5% CO_2_).

### NK cell cytotoxicity assay

K562 or RT4 target cells were labeled with carboxyfluorescein succinimidyl ester (CFSE) (2 μM, Cell Trace CFSE Cell Proliferation Kit, Life Technologies) to discriminate target cells from effector cells (sorted NK cells from human bladder tumors). Effector cells were incubated with CFSE-labeled target cells at different effector-to-target (E:T) ratios in 96-well plates. The cells were cultured in 200 μL culture media, and 10,000-20,000 target cells were used. Heat-killed CFSE-labeled target cells (65°C for 5 minutes and then ice for 1 minute) was used as a positive control. After co-culture for 2.5 hours at 37°C, 5% CO_2_, the cell mixture was stained with 1:1000 dilution of Fixable Viability Dye (FVD) for 30 minutes in the dark and analyzed by flow cytometry. NK cell cytotoxicity (% target cell death) was calculated as cells positive for both CFSE and FVD positive/ total CFSE positive cells. To block the natural-killer group 2, member D (NKG2D) receptor on the NK cells, NKG2D blocking antibody (Biolegend, αNKG2D, 0.01 μg/μl) was added 30 minutes at 37°C prior to the assay. To block MHC I, CFSE labeled target cells were incubated with MHC I blocking antibody (Biolegend, W632, 30 μg/ml) for 30 minutes at 4°C prior to addition of the NK cells.

### Statistical analysis

Pairwise differences in NK cells *in vitro* cytotoxicity were compared using two-sided paired Student's t test. IFN-γ and TNF-α cytokine production, and the proportion of NK cells between any two groups were compared using two-sided unpaired Student's t test. Linear regression was used to test for trend across pathologic stages (<T2, T2, and >T2). The Kaplan-Meier method was used to graph survival across NK cells subsets. The log-rank (Mantel-Cox) test compared survival distributions between groups.

Single variable and multivariable Cox proportional hazards regression models were used to identify associations with cancer-specific survival (CSS), determined by time to death from BC, and overall survival (OS), determined by time to death from any cause. A clinical and demographic Cox model for survival was built by including all such variables (age, gender, stage) that had significant associations with survival and then fitted with the continuous variable NK cells. In all models, proportional hazards assumptions were systematically verified using the Grambsch-Therneau residual-based test. Correlation analyses were carried out using Kendall's tau test. For all analyses, a p-value of < 0.05 was considered statistically significant and all p-values were two-sided. Statistical analyses were performed using Stata/IC 10.1 or GraphPad Prism 6 or R 3.3.0.

## CONCLUSIONS

Intratumoral NK cells are prognostically relevant in BC. However, their association with survival appears to be attributable to a subset of NK cells defined by high surface expression of the CD56 molecule. Bladder intratumoral NK cells exhibit novel characteristics including loss of mutual exclusivity of functional properties with CD56^bright^ NK cells showing both increased cytokine production and cytotoxicity compared to their CD56^dim^ counterparts. Whereas the presence of CD56^bright^ NK cells in bladder tumors is associated with improved survival independent of tumor stage, the presence of CD56^dim^ NK cells portends a worse prognosis by virtue of its strong association with higher tumor stage. Thus, CD56^dim^ NK cells are dysfunctional and could be important targets for improving outcomes for patients with higher stage bladder tumors.

## SUPPLEMENTARY MATERIALS FIGURES AND TABLES



## Abbreviations

NK cells: Natural killer cells
BC: bladder cancer
CSS: cancer-specific survival
OS: overall survival
FACS: fluorescence-activated cell sorting
MHC I: major histocompatibility complex class I
CFSE: carboxyfluorescein succinimidyl ester
FVD: Fixable Viability Dye
NKG2D: Natural Killer Group 2D
MICA: MHC class-I chain related protein A
MICB: MHC class-I-chain related protein B
ULBP1-6: UL16-binding proteins
