# Supplementary material for: Intratumoral CD56bright natural killer cells are associated with improved survival in bladder cancer
Source: Oncotarget. 2018 Nov 23;9(92):36492–502. doi: 10.18632/oncotarget.26362 (PMC6284861; doi:10.18632/oncotarget.26362)
Supplement: Supplementary file 1 [file oncotarget-09-36492-s001.pdf]

Intratumoral CD56[bright] natural killer cells are associated with improved survival in bladder cancer

SUPPLEMENTARY MATERIALS

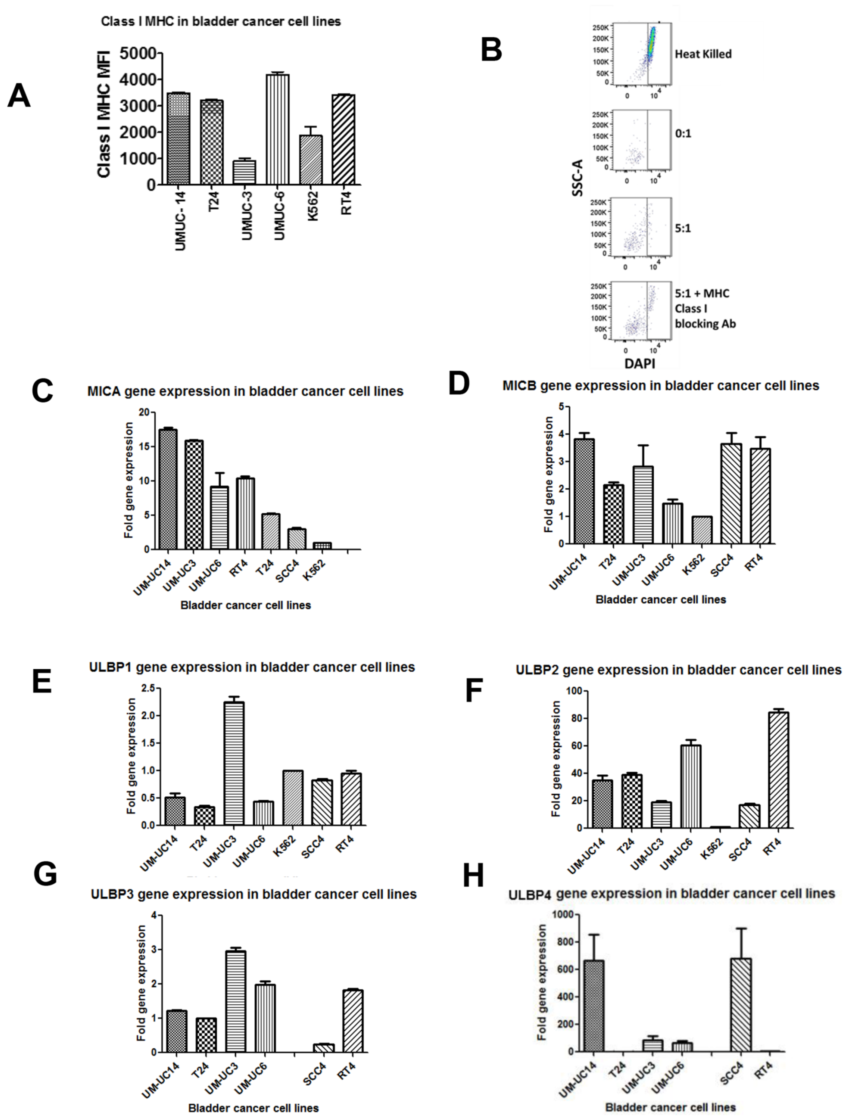

Supplementary Figure 1: Expression of NK cell ligands on human cancer cells. (A) Human cancer cell lines (UMUC-14, T24, UMUC-3, UMUC-6, K562, and RT4) were cultured and processed for staining of Class I MHC by flow cytometry. Mean ± SEM. **(B)** Representative flow gating of NK cell cytotoxicity of NK cell cytotoxicity assay. Sorted NK cells from human bladder tumor assayed with CFSE-labeled RT4 bladder cancer target cells. The positive control was heat killed CFSE-labeled target cells and negative control was live CFSE labeled target cells alone. To block, MHC I CFSE labeled target cells were incubated with MHC I blocking antibody. Human cancer cell lines (UMUC-14, T24, UMUC-3, UMUC-6, K562, and RT4) were cultured and RT-PCR is carried out to test the mRNA expression of **(C)** MHC class I polypeptide-related sequence A (MICA), **(D)** MHC class I polypeptide-related sequence B (MICB), **(E)** UL16 binding protein 1 (ULBP1), **(F)** UL16 binding protein 2 (ULBP2), **(G)** UL16 binding protein 3 (ULBP3), **(H)** UL16 binding protein 4 (ULBP4). Mean ± SEM.

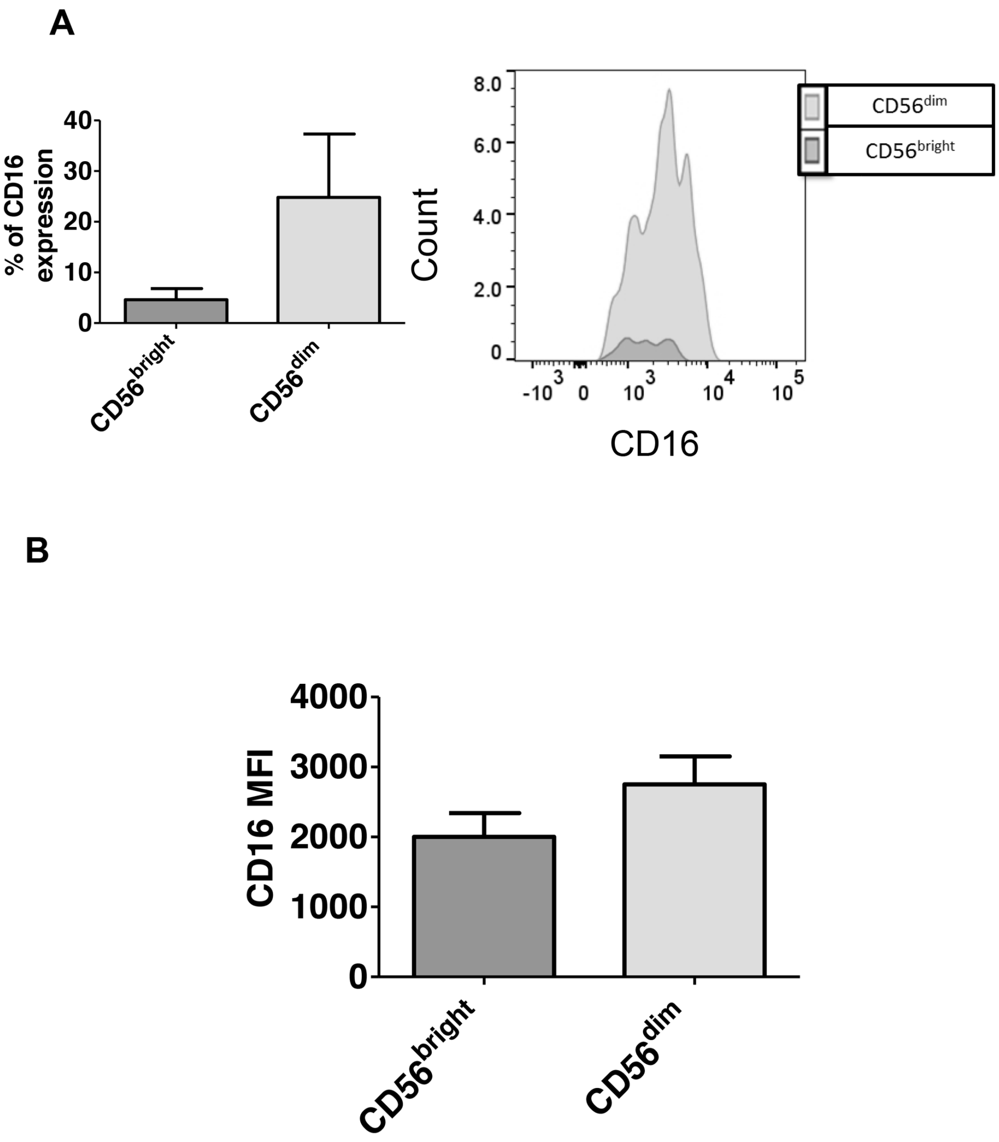

Supplementary Figure 2: CD16 expression is lower on CD56^{bright} compared to CD56^{dim} bladder intratumoral NK cells. Human bladder tumor samples from patients with bladder cancer were processed into single cell suspensions and NK cells were identified as live CD45^{+} Lin (CD14, ILT3, CD19)$^{-}$CD3^{-} cKIT^{-} cells and were further characterized into CD56^{bright} and CD56^{dim} populations. **(A)** Graph of percent of CD16 on CD56^{dim} and CD56^{bright} NK cells. **(B)** Density of CD16 surface expression evaluated by mean fluorescence intensity (MFI) were further determined on CD56^{dim} and CD56^{bright} NK cells populations by flow cytometry.

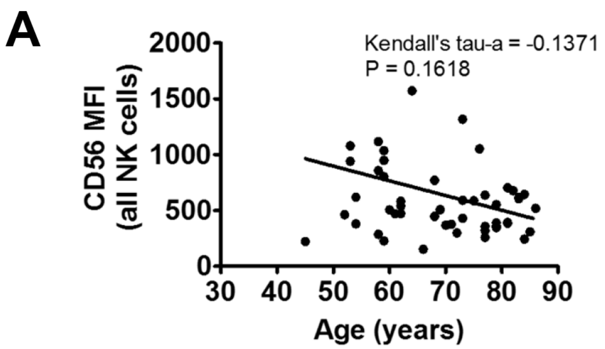

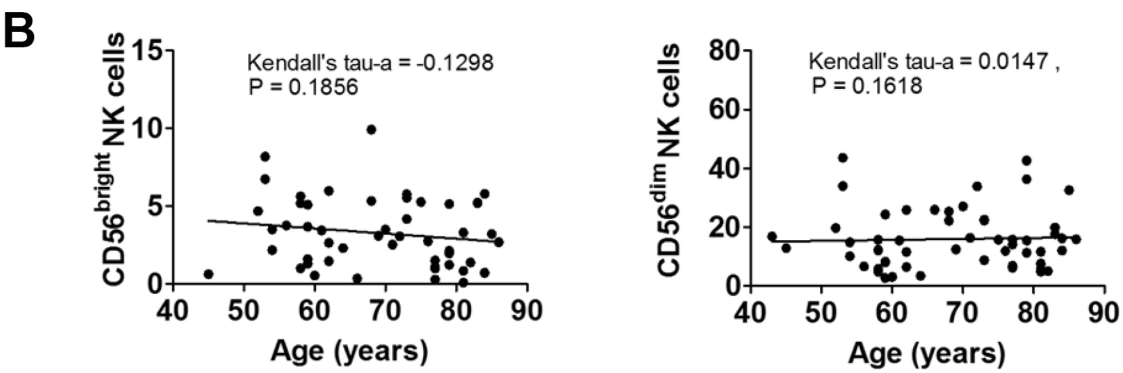

Supplementary Figure 3: Association of NK cell subsets with aging in local bladder cancer patient cohort. (A) Bladder tumor samples from patients (n=50) with BC were processed into single cell suspensions and NK cells were identified as live $CD45^{+}$ Lin (CD14, ILT3, CD19)$^{-}$ $CD3^{-}$ $cKIT^{-}$ cells. Scatterplot with linear trend line of CD56 mean fluorescence intensity (MFI) on NK cells across patient age is plotted (Kendall's tau-a = -0.1371, P = 0.1618). **(B)** NK cells were further characterized into $CD56^{bright}$ and $CD56^{dim}$ subsets and scatterplots with linear trend line of the percentage of these two subsets across patient age are plotted.
